# Supplementary material for: Factors Associated with Depression and Anxiety Symptoms among Migrant Population in Spain during the COVID-19 Pandemic
Source: Int J Environ Res Public Health. 2022 Nov 24;19(23):15646. doi: 10.3390/ijerph192315646 (PMC9736595; doi:10.3390/ijerph192315646)
Supplement: Supplementary file 1 [file ijerph-19-15646-s001.zip › ijerph-1950049-supplementary.pdf]

Table S1. Individual characteristics.

	Models					
Variables	Depressive symptoms			Anxiety symptoms		
	IRR	SE	p-value	IRR	SE	p-value
(Intercept)	4.5530	1.1670	0.0000	6.7760	1.7750	0.0000
G3_resilience	1.1810	0.0690	0.0050	1.1440	0.0700	0.0260
M21_selfEsteem	1.0660	0.0100	0.0000	1.0410	0.0100	0.0000
A1	0.9760	0.0050	0.0000	0.9730	0.0050	0.0000
A2Femenino	1.0520	0.0910	0.5600	1.0440	0.0920	0.6230
A2Otro	0.0000	0.0000	0.9830	0.0000	0.0000	0.9830
Num.Obs.	93			93		
AIC	658.9			621.0		
BIC	674.1			636.2		
Log.Lik.	-323.454			-304.479		
F	18.749			11.858		
RMSE	4.69			4.30		

Table S2. Factors related to the migration process.

	Models					
Variables	Depressive symptoms			Anxiety symptoms		
	IRR	SE	p-value	IRR	SE	p-value
(Intercept)	15.6710	2.6490	0.0000	14.7500	2.5230	0.0000
M2DichCon permiso de residencia	1.1460	0.1160	0.1780	1.1680	0.1210	0.1330
A1	0.9700	0.0050	0.0000	0.9690	0.0050	0.0000
A2Femenino	0.9850	0.0890	0.8660	0.9930	0.0920	0.9430
A2Otro	0.0000	0.0000	0.9830	0.0000	0.0000	0.9830
comunitarioComunitario	2.0270	0.3470	0.0000	1.9400	0.3500	0.0000
comunitarioOtro	0.7210	0.0990	0.0170	0.9540	0.1200	0.7110
Num.Obs.	93			93		
AIC	685.0			626.4		
BIC	702.8			644.1		
Log.Lik.	-335.521			-306.203		
F	10.837			9.109		
RMSE	4.96			4.40		

Table S3. Factors related to basic needs (housing and employment).

	Models					
Variables	Depressive symptoms			Anxiety symptoms		
	IRR	SE	p-value	IRR	SE	p-value
(Intercept)	11.1080	2.6970	0.0000	13.7590	3.1680	0.0000
M12No	1.1530	0.1010	0.1060	1.4240	0.1300	0.0000
M15TrichAlquiler	1.2960	0.2150	0.1180	0.9190	0.1350	0.5630
M15TrichSin piso	1.2050	0.2350	0.3370	0.7760	0.1400	0.1600
A1	0.9720	0.0050	0.0000	0.9710	0.0050	0.0000
A2Femenino	1.0620	0.0920	0.4870	1.0360	0.0920	0.6910
A2Otro	0.0000	0.0000	0.9830	0.0000	0.0000	0.9830
Num.Obs.	93			93		
AIC	705.8			625.9		
BIC	723.5			643.6		
Log.Lik.	-345.878			-305.938		
F	7.049			9.119		
RMSE	5.09			4.35		

Table S4. Factors related to the social environment.

	Models					
Variables	Depressive symptoms			Anxiety symptoms		
	IRR	SE	p-value	IRR	SE	p-value
(Intercept)	66.7330	50.4750	0.0000	56.5240	37.2340	0.0000
HOSLOv_socialSupport	0.9280	0.0190	0.0000	0.9840	0.0200	0.4430
G1	0.2460	0.1310	0.0080	0.2930	0.1300	0.0060
M22_everydayDS1	0.6330	0.4700	0.5380	0.5460	0.3500	0.3450
A1	0.9770	0.0050	0.0000	0.9750	0.0050	0.0000
A2Femenino	0.9880	0.0870	0.8890	0.9600	0.0870	0.6540
A2Otro	0.0000	0.0000	0.9840	0.0000	0.0000	0.9840
G1 × M22_everydayDS1	2.6870	1.4320	0.0640	2.3020	1.0290	0.0620
Num.Obs.	93			93		
AIC	596.5			561.2		
BIC	616.8			581.5		
Log.Lik.	-290.255			-272.622		
F	18.256			14.334		
RMSE	4.42			4.05		

Table S5. Perceived health.

	Models					
Variables	Depressive symptoms			Anxiety symptoms		
	IRR	SE	p-value	IRR	SE	p-value
(Intercept)	25.0780	6.5340	0.0000	37.4490	9.8070	0.0000
EQ5D5L_crosswalk	0.3310	0.0700	0.0000	0.2620	0.5060	0.0000
H11Si	1.2590	0.1110	0.0090	1.1300	0.1040	0.1840
C1Si	1.2630	0.1250	0.0180	1.0170	0.1010	0.8650
A1	0.9780	0.0050	0.0000	0.9760	0.0050	0.0000
A2Femenino	1.0740	0.0940	0.4150	1.0690	0.0950	0.4550
A2Otro	0.0000	0.0000	0.9840	0.0000	0.0000	0.9830
Num.Obs.	93			93		
AIC	655.4			599.0		
BIC	673.1			616.7		
Log.Lik.	-320.700			-291.481		
F	16.441			14.982		
RMSE	4.75			4.22		
